# Discharged. Not Where to Lay His Head

**Published:** 1886-10-30

**Authors:** George Fleming


### DISCHARGED.

### "NOT WHERE TO LAY HIS HEAD."

By George Fleming.

VI.

When Lemon lounged downstairs in the morning, the church bells were already ringing far and near. The family, collected in the front entrance, were already dressed for church, and,—"Lord! if here isn't one of them that I had clean forgotten!" his hostess cried out, a sudden sharp vexation clouding all her rosy face.

Lemon apologised. "I had a bad night. But if you'll believe me, I had no idea it was so late," he said, with his conciliatory smile.

"Oh—ideas! Arn't there watches? It ain't a cook some folks needs, but a saint in their kitchens," quoth the good woman briefly. She glanced at her daughter's flat waist and shoulders tightly squeezed into a jacket of brillant blue velveteen, and then at her own gloved fingers.

"There's no one left in the house but that boy, and who's to cook breakfast for you *I* don't know," she continued, after a severe pause of condemnation; "lyin' in bed is one thing, and I ain't one of those who takes my Sunday morning in a skurry. The day of rest is *for* rest; that's what I say. But there's public-houses where they ain't shut up yet." She hesitated, gathering her Paisley shawl more firmly over her substantial shoulders. "Temperance principles is one thing. But it's eleven o'clock in the morning, and the kitchen fire out since nine," she said at last with recovered firmness. "And let me warn you, young man, not to think of going out on a day like this, with your cough, unless you carry an umbrella." She came back from the front door to say this.

It was indeed a dismal breaking up of the golden weather. Lemon drank his coffee, standing, on the corner of a counter over which spirits and beer were being sold. Every minute or two the swinging door behind him was pushed open; some dripping figure stepped in, laid down its money and tossed off a corresponding dram. Man or woman, young or old, exuberant with coarse animal life, or white-cheeked, with wild, haggard eyes—the ghastly procession never stopped for one moment, coming in from the street; going back to the street. Now and then some of the younger men who came in small squads would stop to talk for a little; but most of them, and all the women, were silent.

It was too early yet in the day for noise and boisterous laughter; these were merely the people who drink as a matter of course,—to cheat memory once more, and lift for a moment, if it may be, the dull pressure of want and discouraged effort which makes the stuff of their daily lives.

Lemon lingered there so long that at last one of the attendants came up and spoke to him.

"Must close now for an hour or two. I've a particular engagement at the nearest church myself," he said, winking with cheerful derision. "It's some time since you 'ad that coffee; you wouldn't take a drop o' something else, perhaps, just to keep out the chill before you go?"

"No; no, thank'ee. You see, it is not in my line," Lemon answered, doubtfully.

"Well, if you won't, you won't. There's plenty more that will," the shopman retorted with perfect good-humour.

He turned away to fasten the side-door, and Lemon walked out into the wet street. Some idea of going down to Rotherhithe,—not to see anyone, for this was not the day on which visitors were admitted, but some vague wish to find himself in a place with which he had associations, crossed his mind. It was of no use going to see Mary Parker; her aunt was making the best of their holiday, and the two women would spend Sunday listening to the eloquence of Mr. Spurgeon. There was his old ally, the ex-Chartist, but Lemon only knew of him vaguely as living somewhere in the Marylebone Road; and how was he to go and find him out in this black torrent of rain? After a minute or two of hesitation, it ended by his making the best of his way back to the little empty bedroom under the roof.

Perhaps he had caught cold last night in the Park. There was no telling. But his chest felt burning and sore, and he missed the care to which he had grown accustomed. He missed the doctor's authority and the cheerful decision of the nurse. If there had only been some other place for him to go—something different from this dark, sordid room, made still more unattractive by the tossed and draggled heap of bedclothes. A horrible, deadly, leaden *ennui* seemed to drip, drip with the rain from out that drowned sky. He took his dog upon his knee and sat down on the foot of the disordered bed, fixing his eyes upon the glimmering square of window. It was an hour of great loneliness—that passion of the spirit's loneliness which can only befall a sensitive and imaginative nature, and which has made great artists before now and evoked immortal poems. But Nature, unfortunately for us poor mortals, forces the same overmastering passions through widely differing human channels. This black hour brought to Lemon no compensating power of expression; to him only a duller sense of misery seemed to ooze out of every crack between the bricks in the soot-blackened wall opposite which bounded his horizon.

He began, after a while, to think about the gilding job that was coming to him on Tuesday. He had been used to take the satisfaction in his work which goes with skilful hands, but now, after all these months and months of stupefying inaction, it was difficult to realise the pleasure of energetic action, and Tuesday seemed a long way off.

It only made his chest feel worse to sit there doing nothing. After a time, for lack of other amusement, he got out his pledge-card and began carving a frame for it, with his pocket-knife, from a piece of soft wood which Spot had been playing with in the corner of the room. When dinner time came he seemed to have lost all sensation of hunger. The greasy smell of roasting mutton, which steamed up the stairs from the kitchen, only made him realise more keenly how difficult it would be to eat. He counted over the change he had left from Dick's half-sovereign, and it did not seem a bad plan to put off feeding until night; still he had remembered to put some bread in his pocket for Spot. He broke it up now into little pieces and tried to get the dog to stand on his hind paws and beg.

He worked at his frame until dark. When he could no longer see, he threw himself down on the bed with the intention of sleeping; but he had not been lying there ten minutes when he started up again, springing suddenly to his feet as if some one had called him. Anyone who could have seen him at that moment must have been struck with the singular expression of his face. His eyes had the fixed, abstract gaze you may see in the dilated pupil of some caged wild beast; and no hungry animal, scenting blood, could have moved with a more furtive precision. He snatched up his hat and walked straight out of the room, Spot following close behind him. At the bottom of the dark stairway he turned noiselessly to the left, to avoid passing through the coffee-room, where the gas was already lighted, and whence issued a loud hum of voices.

It was raining harder than ever, but he moved as deliberately as if the rain and wind had no longer an existence for him. Two or three men, with pipes in their mouths, were coming out of the public-house where he had bought his breakfast that morning. One of them said something as he passed, but Lemon took no notice of it. He pushed open the green swinging door and walked straight up to the shining counter. "Fourpennyworth of brandy and water—hot," he said, automatically.

The strong spirit sent the blood coursing lightly and pleasantly through every vein of his enfeebled body. The dull, unbearable sense of oppression which had weighed him down all day was lifted as if by magic. He felt himself his own man again, and he tramped back cheerfully through the dark, wet streets, whistling like a bird out of very lightness of heart.

*(To be continued.)*

